# Supplementary material for: SSEA3 and Sialyl Lewis a Glycan Expression Is Controlled by B3GALT5 LTR through Lamin A-NFYA and SIRT1-STAT3 Signaling in Human ES Cells
Source: Cells. 2020 Jan 10;9(1):177. doi: 10.3390/cells9010177 (PMC7017369; doi:10.3390/cells9010177)
Supplement: Supplementary file 1 [file cells-09-00177-s001.pdf]

Supplemental Information

SSEA3 and sialyl Lewis a glycan expression is controlled by B3GALT5 LTR through lamin A-NFYA and SIRT1-STAT3 signaling in human ES cells

Bi-He Cai, Hsueh-Yi Lee, Chi-Kan Chou, Po-Han Wu, Hsiang-Chi Huang, Chia-Chun Chao, Hsiao-Yu Chung and Reiji Kannagi

Supplemental Figures

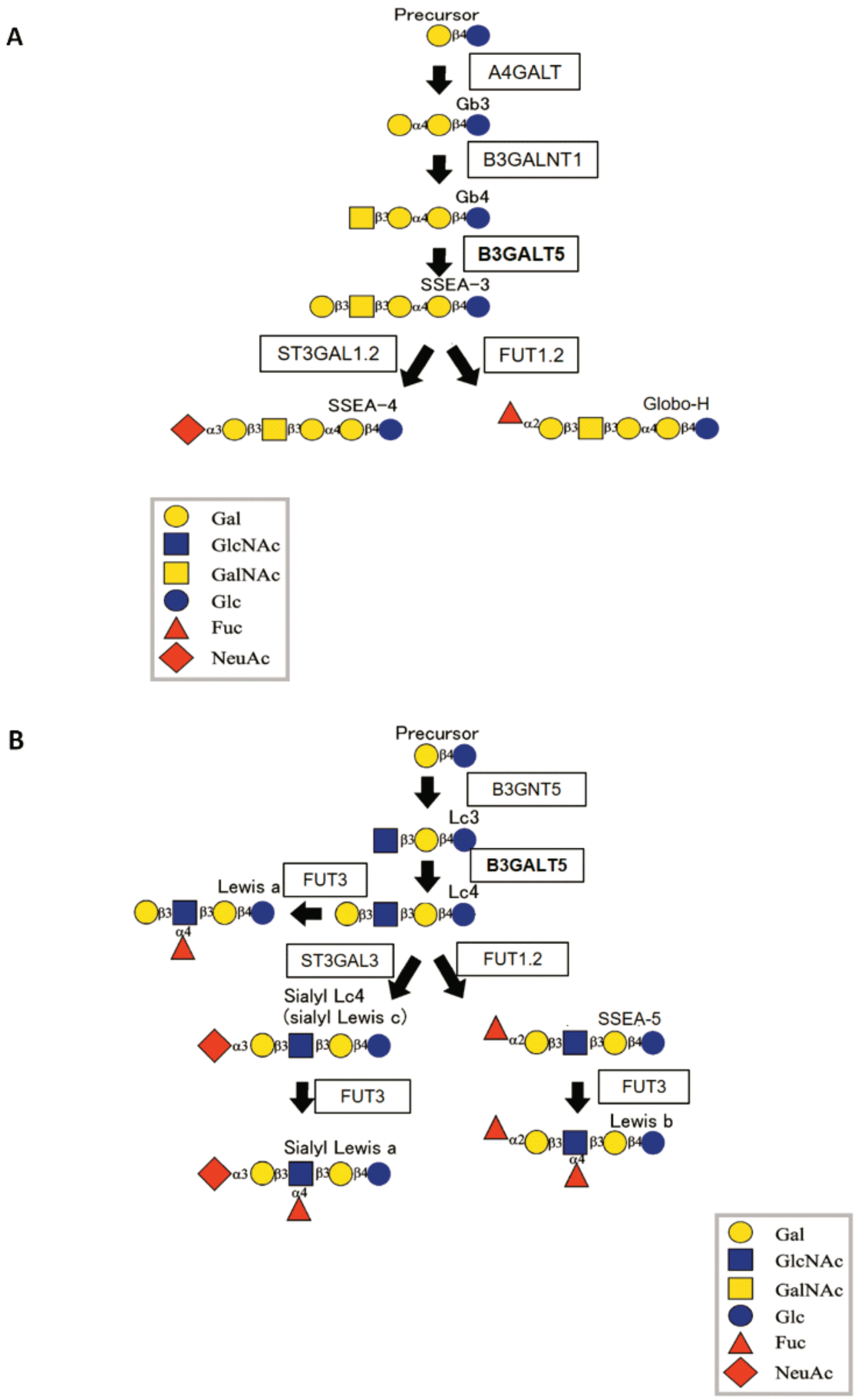

Fig. S1. Involvement of *B3GALT5* activity in glycan synthesis. (**A**) Biosynthetic pathways for globoseries glycolipids. (**B**) Biosynthetic pathways for type-1-chain lactosamine glycans.

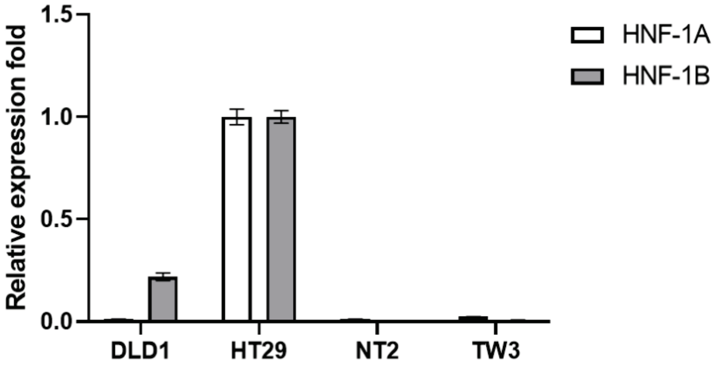

Fig. S2. HNF-1 is highly expressed in colon cancer cells but not EC and ES cells.

The levels of HNF-1A and 1B in HT29 cells were normalized to 1. HNF-1A was nearly absent in DLD1, NT2, and TW3 cells. HNF-1B was expressed in DLD1 but not EC or ES cells (n = 3 per group).

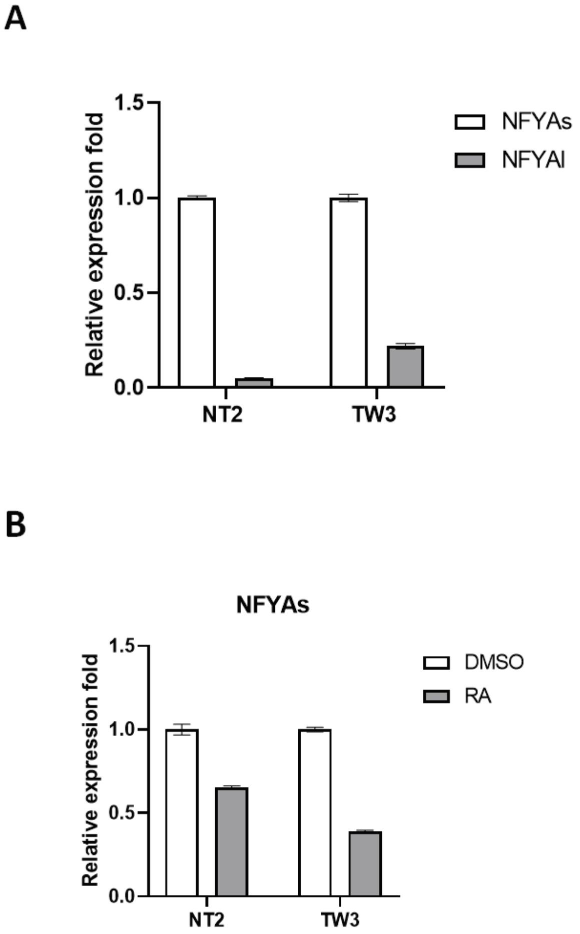

Fig. S3. Reduction of NFYAs in differentiated EC and ES cells. (**A**) NFYAs is more highly expressed than NFYAl in NT2 and TW3 cells (n = 3 per group). (**B**) NFYAs expression is reduced in differentiated NT2 and TW3 cells (n = 3 per group).

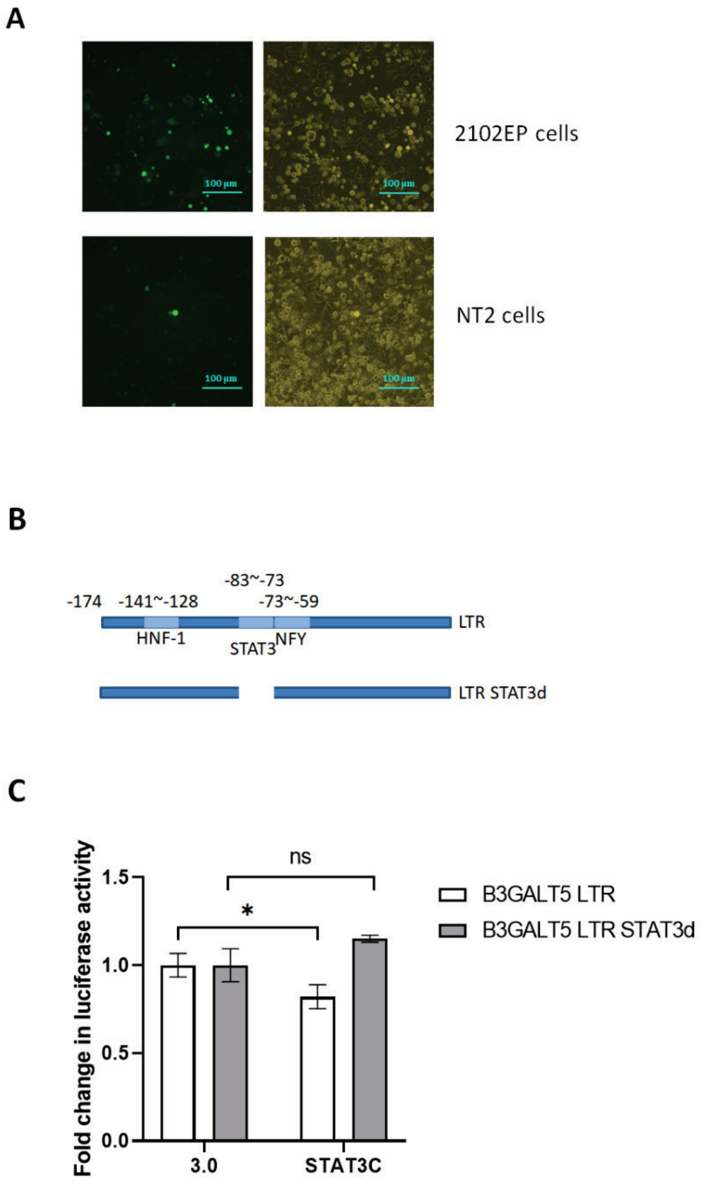

Fig. S4. STAT3C represses *B3GALT5*-LTR promoter activity in 2102Ep cells.

(**A**) Transfection efficiencies of 2102Ep and NT2 cells. GFP fluorescence served as an indicator of transfection efficiency, which was greater in 2102Ep cells than in NT2 cells. (B) The positions of the canonical HNF1-, STAT3- and NFY-binding sites on the *B3GALT5*-LTR promoter are shown. (C) Expression of STAT3C reduces the *B3GALT5*-LTR promoter reporter response, and this response is rescued by deletion

of the canonical STAT3-binding site on the *B3GALT5*-LTR promoter in 2102Ep cells (n = 3 per group).

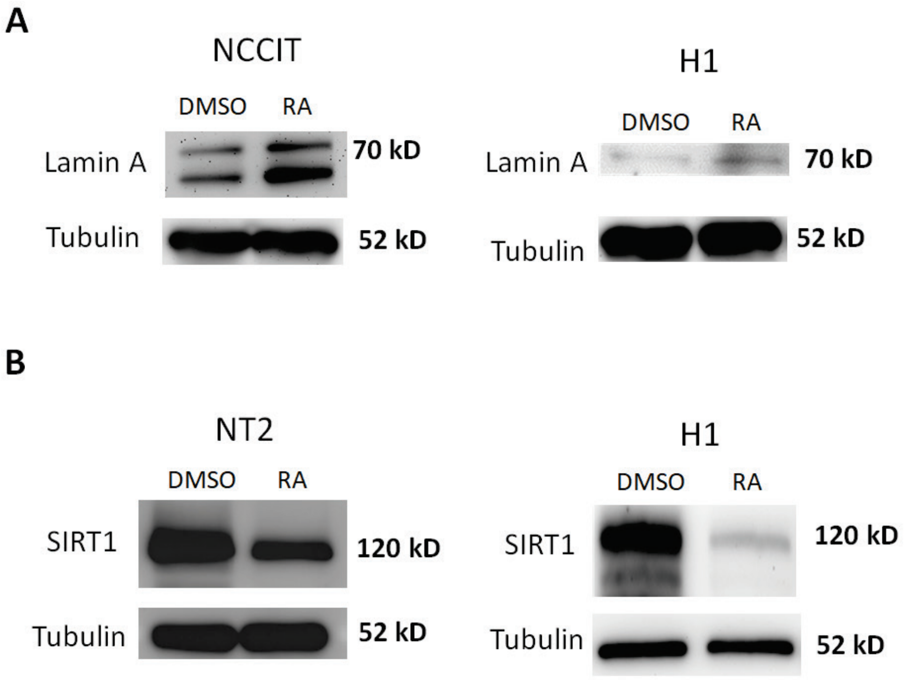

Fig. S5. Treatment of cells with RA alters the levels of SIRT1 and lamin A. (**A**) Lamin A level increases in NCCIT and H1 Oct4-EGFP cells after 1-week treatment with RA. (**B**) SIRT1 level decreases in NT2 and H1 Oct4-EGFP cells after 1-week treatment with RA.

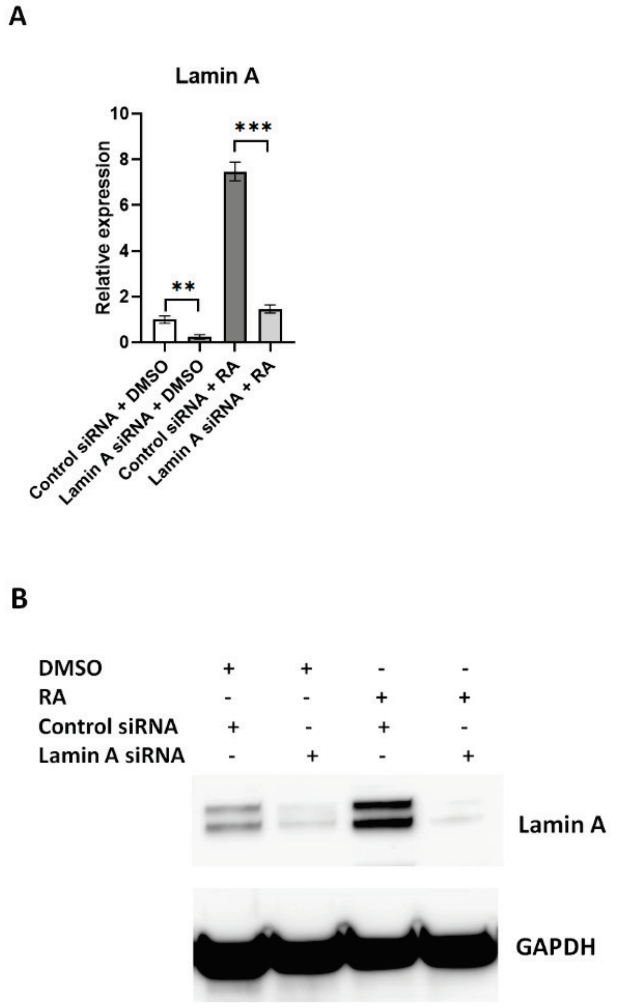

Fig. S6. Lamin A expression pattern in RA- or DMSO-treated NT2 cells with siRNA(s) transfection. (**A**) The mRNA of lamin A level increased after RA treatment but was significantly reduced upon treatment of cells with lamin A–specific siRNA compared with control siRNA in DMSO- and RA-treated cells (n = 3 per group). (B) The protein level of lamin A increased after RA treatment but was significantly reduced upon treatment of cells with lamin A–specific siRNA.

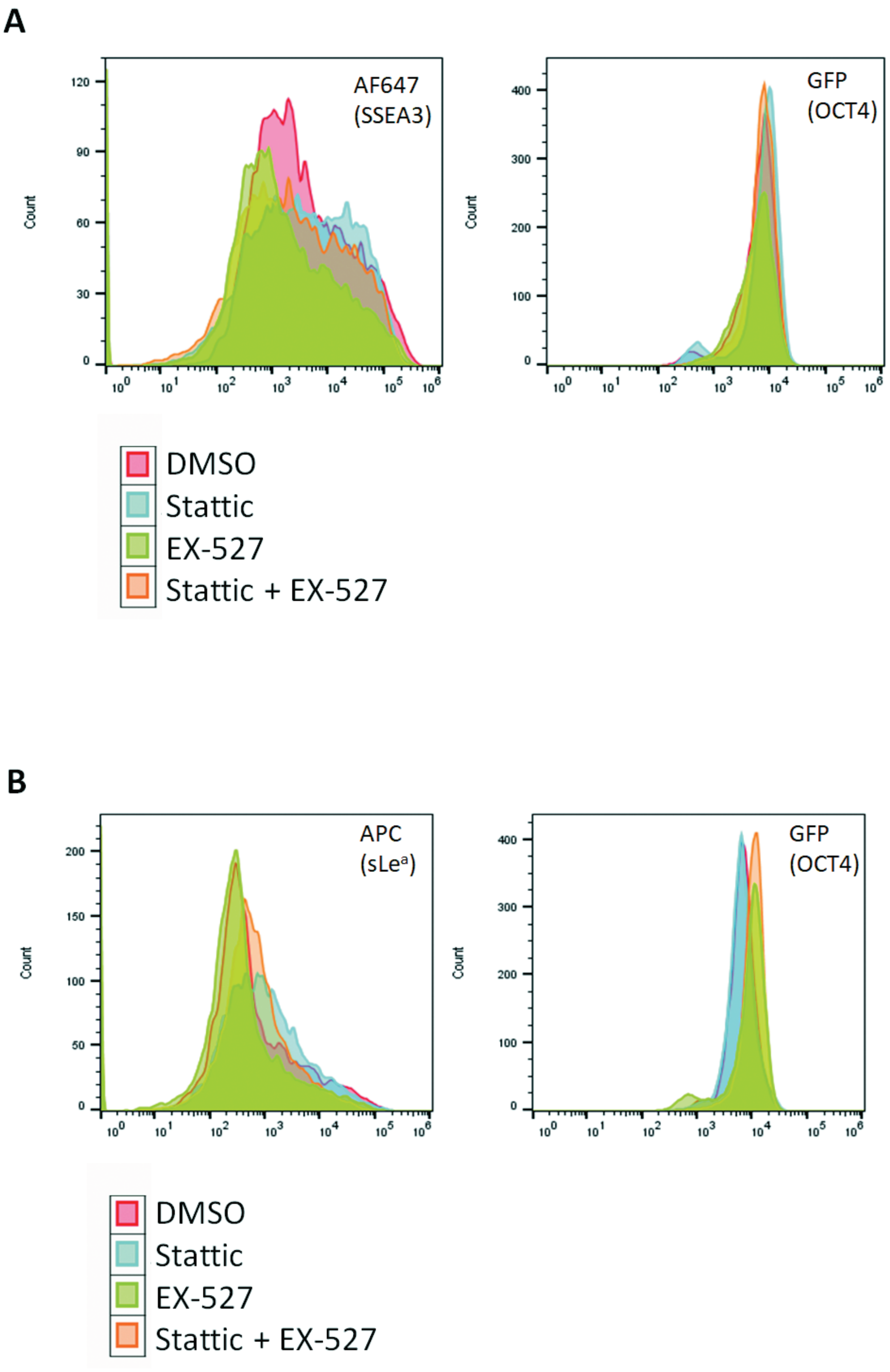
A
AF647
(SSEA3)
GFP
(OCT4)
Count
DMSO
Stattic
EX-527
Stattic + EX-527
B
APC
(sLea)
GFP
(OCT4)
Count
DMSO
Stattic
EX-527
Stattic + EX-527

Fig. S7. EX-527 represses SSEA3 and sialyl Lewis a expression in H1 Oct4-EGFP cells, and this repression is partially reversed by Stattic. (**A**) EX-527 represses SSEA3 synthesis in H1 Oct4-EGFP cells, and this repression is partially reversed by Stattic. (**B**) EX-527 represses sialyl Lewis a synthesis in H1 Oct4-EGFP cells, and this repression is partially reversed by Stattic.

A

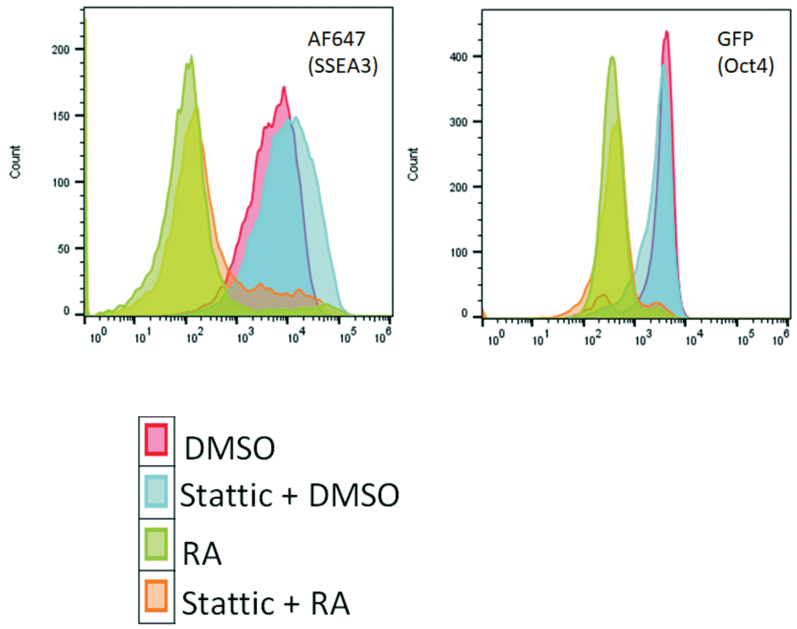
AF647
(SSEA3)
Count
GFP
(Oct4)
DMSO
Stattic + DMSO
RA
Stattic + RA

B

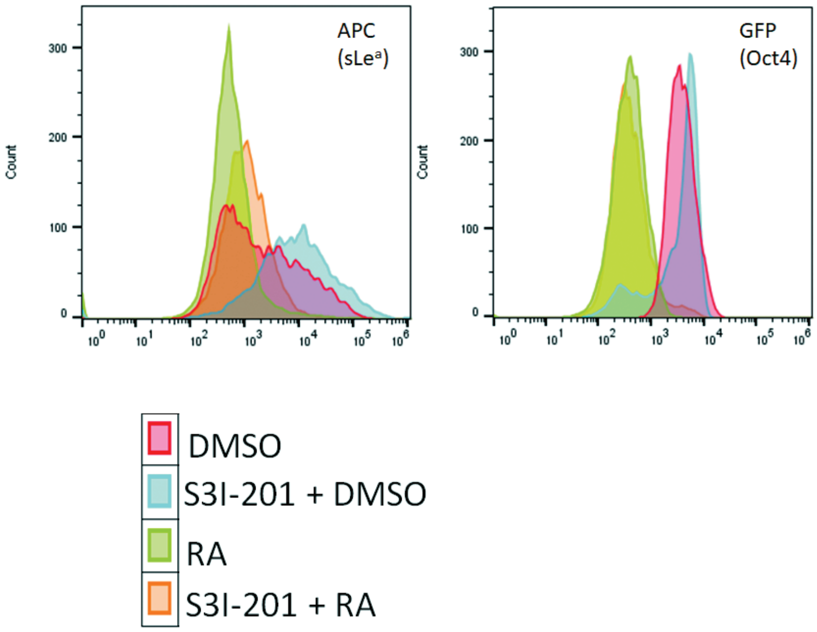
APC
(sLe^a)
Count
GFP
(Oct4)
DMSO
S3I-201 + DMSO
RA
S3I-201 + RA

Fig. S8. RA represses SSEA3 and sialyl Lewis a expression in H1 Oct4-EGFP cells, and this repression is partially reversed by STAT3 inhibitor. (**A**) RA represses SSEA3 synthesis in H1 Oct4-EGFP cells, and this repression is partially reversed by Stattic. (**B**) RA represses sialyl Lewis a synthesis in H1 Oct4-EGFP cells, and this repression is partially reversed by S3I-201.

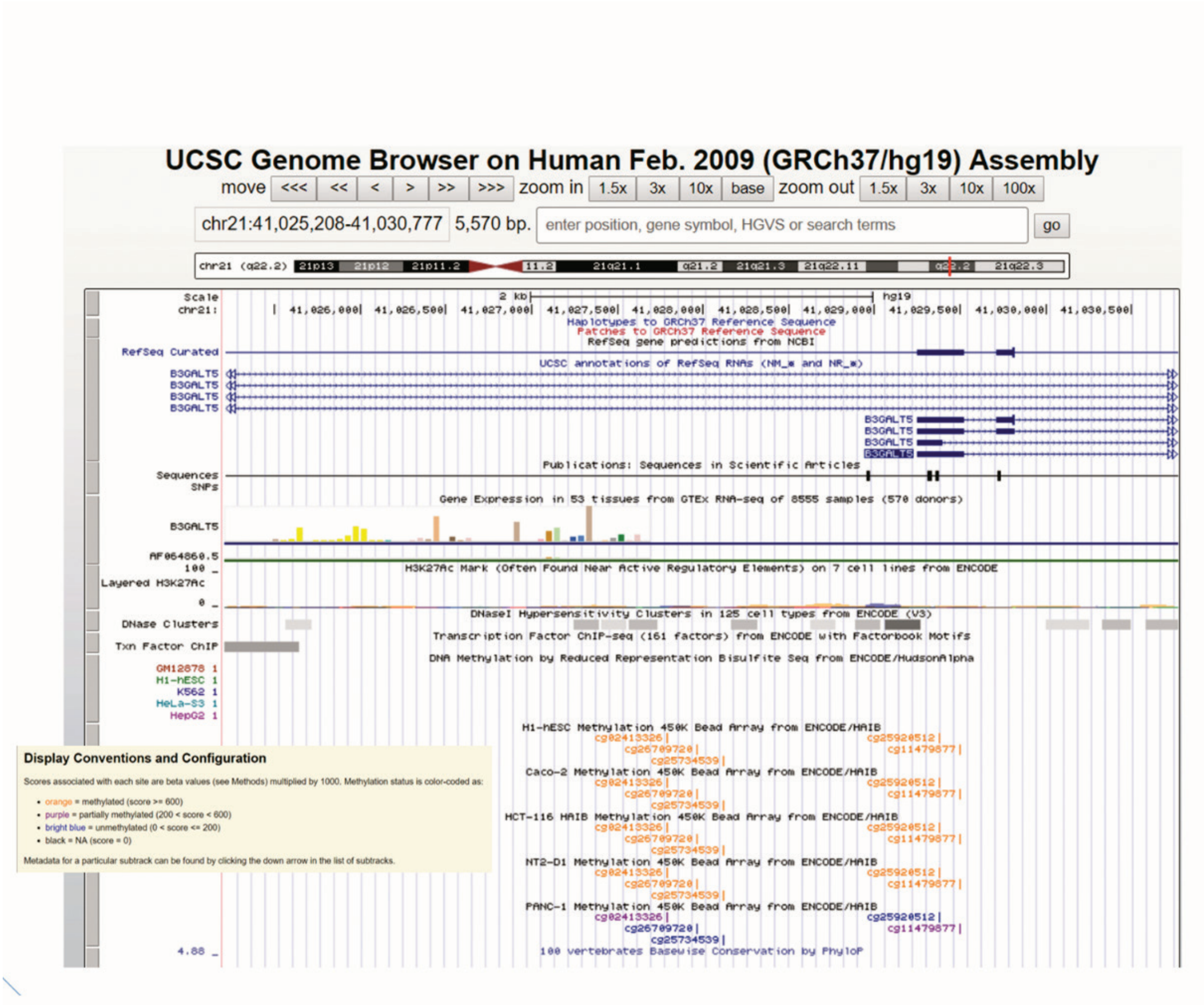

Fig. S9. The methylation map from the UCSC Genome Browser database showing the upstream *B3GALT5*-LTR exon. Sites 1 and 2 containing CpG islands located on the upstream of B3GALT5-LTR exon are highly methylated in EC cells (NT2), ES cells (H1), and colon cancer cells (Caco-2 and HCT-116) but are unmethylated or only partially methylated in pancreatic cancer cells (PANC-1).

Supplemental table

Table S1. Primers used for conventional qPCR.

For cDNA amplification

Gene	Primer sequence (5′–3′)	Length, bp	Annealing temperature, °C
GAPDH	F: GTCTCCTCTGACTTCAACAGCG	131	60
	R: ACCACCCTGTTGCTGTAGCCAA		60
B3GALT5-LTR	F: GGCCCAGATAGAACAAGAAGG	133	60
	R: GGTATAAATCCTCGGGTCCAA		60
Total B3GALT5	F: TGGGTCCATCGCTTTTGTCC	160	60
	R: GCTGCCTGATGGGAAACTCA		60
B3GALT5-Type A transcripts	F: ATCCTCGGCCTGGACCC	77	60
	R: CTCAGAGCCGCATCCCG		60
B3GALT5-Type B transcripts	F: GAAACCCAAATGTTGGAGGGG	82	60
	R: GTGTGGCCACGAGTGTCAAA		60
B3GALT5-Type C transcripts	F: AGGAATGGCTGAGCTGAGAAG	66	60
	R: TGCTTTGTCTATGCAGGTCCC		60
HNF-1A	F: GCGTCTACCCTGGGATTCAG	123	60
	R: TCCACCGCATTTCTCCTTGA		60
HNF-1B	F: AACACAACATCCCCCAGAGG	180	60
	R: CAGAACTCTGGACTGTCTGGT		60

NFYAs	F: ACAGATTCAGCAGCAGGTCC	84	60
	R: ATGGGTTGGCCAGTTGATGT		60
NFYAl	F: CTGTGCAGTTGCAGACTGAGG	160	60
	R: CTCCACTGACCTGCACCATTA		60
Lamin A	F: CTGTGGTTGAGGACGACGAG	240	60
	R: TGCGGTAGCTGCGAGTGA		60

For ChIP assays

Gene	Primer sequence (5′–3′)	Length (bp)	Annealing temperature °C
B3GALT5-LTR promoter	F: TCTCTCCTGGCATCTATATTGCT	172	60
	R: GCCCACTGACTCACAGCCAATCT		60
B3GALT5-ORF	F: CGTGGGGGAAAGAGAGGATG	124	60
	R: ATAATGTCCCCGTGTCGCTG		60
CRM1 promoter	F: GTGAGGCGGGATTGACTG	264	60
	R: TGCTGATGCTGTAGCTCCC		60
CASP3 promoter	F: TCCCAACAGCCGGCTTAA	66	60
	R: AAGAAGCCTGGTTTGGG		60
